# Prognostic impact of peripheral blood *WT1-*mRNA expression in patients with MDS

**DOI:** 10.1038/s41408-019-0248-y

**Published:** 2019-11-12

**Authors:** Christina Rautenberg, Ulrich Germing, Sabrina Pechtel, Marius Lamers, Carolin Fischermanns, Paul Jäger, Stefanie Geyh, Rainer Haas, Guido Kobbe, Thomas Schroeder

**Affiliations:** Department of Hematology, Oncology and Clinical Immunology, University of Duesseldorf, Medical Faculty, Duesseldorf, Germany

**Keywords:** Myelodysplastic syndrome, Cancer genetics

## Abstract

Few reports suggested a prognostic impact of *Wilms‘Tumor-1* (*WT1*)-mRNA overexpression in MDS, but translation into clinical routine was hampered by limited patients numbers, differing sample sources, non-standardized methods/cut-offs. To evaluate whether *WT1-*mRNA expression yields additional prognostic information, we measured peripheral blood (PB) *WT1-*mRNA expression in 94 MDS using a standardized assay offering a validated cut-off to discriminate between normal and *WT1-*mRNA overexpression. Overall, 54 patients (57%) showed *WT1-*mRNA overexpression, while 40 patients (43%) had normal *WT1*-mRNA expression. This enabled discrimination between MDS and both healthy controls and non-MDS cytopenias. Furthermore, *WT1-*mRNA expression correlated with WHO 2016 subcategories and IPSS-R as indicated by mean *WT1-*mRNA expression and frequency of *WT1*-mRNA overexpressing patients within respective subgroups. Regarding the entire group, PB *WT1-*mRNA expression was associated with prognosis, as those patients showing *WT1-*mRNA overexpression had higher risk for disease progression and AML transformation and accordingly shorter progression-free, leukemia-free and overall survival in univariate analysis. In multivariate analysis, prognostic impact of PB *WT1-*mRNA expression status was independent of IPSS-R and enabled more precise prediction of PFS, but not OS, within IPSS-R very low/low and intermediate risk groups. Overall, measuring PB *WT1-*mRNA appears valuable to support diagnostics and refine prognostication provided by the IPSS-R.

## Introduction

Myelodysplastic syndromes (MDS) are a heterogeneous group of hematopoietic stem cell disorders characterized by hematopoietic insufficiency, variable signs of dysplasia and accumulation of immature precursors in the bone marrow (BM). The course of disease is highly variable and ranges from indolent appearance with almost normal life expectancy to conditions with rapid progression to a more advanced subtype or even acute myeloid leukemia (AML)^1–4^. To estimate the individual risk for disease progression, AML transformation and survival time scoring systems such as the international prognostic scoring system (IPSS) and its revised version (IPSS-R) have been established and are based on clinical variables such as cytopenias, BM blasts, and cytogenetics^5,6^. Taking advantage of the knowledge about several gene mutations recently unraveled in MDS the International Working Group for the Prognosis of MDS (IWG-PM) and others are currently trying to incorporate these in order to improve risk assessment^7–10^. Since this is still work in progress and standardized analyses and reporting of gene mutations is still lacking, we hypothesized that mRNA expression of the *Wilms tumor 1 (WT1)* gene may offer additional prognostic information in patients with MDS based on the following considerations: (1) Overexpression of *WT1*-mRNA has been reported in the about 50% of MDS patients and seems to correlate with disease stage and IPSS risk category^11,12^. (2) A limited number of reports has suggested a prognostic impact of *WT1*-mRNA expression patients with MDS, but their interpretation and translation into clinical routine are hampered by differing sample sources, non-standardized methods and cut-offs^13–16^. (3) A standardized, European Leukemia Net (ELN)-certified assay is available and enables measurement of *WT1*-mRNA expression in peripheral blood (PB) with a reproducible and validated cut-off to distinguish between normal and overexpression of *WT1-*mRNA^17^.

We here used this standardized assay to address the prognostic impact of PB *WT1*-mRNA expression in a cohort of 94 patients covering all common MDS subtypes.

## Patients and methods

### Patients and study design

Ninety-four patients (median age: 61.5 years, range 22–84 years) with newly diagnosed, treatment-naive MDS and available information about the peripheral blood (PB) *WT1*-mRNA expression level at the time diagnosis were included into this retrospective analysis. Among these, three patients (3%) suffered from MDS with single lineage dysplasia (MDS SLD) according to the World Health Organization (WHO) 2016 classification, one patient (1%) from MDS with ring sideroblasts and single lineage dysplasia (MDS RS SLD), 39 patients (41%) from MDS with multilineage dysplasia (MDS MLD), 7 patients (7%) from MDS del(5q), 3 patients (3%) from MDS unclassifiable (3%), 16 patients (17%) from MDS with excess blasts 1 (MDS EB1) and 25 patients (27%) from MDS with excess blasts 2 (MDS EB2). According to IPSS-R 3 (3%), 28 (30%), 28 (30%), 14 (15%), 21 (22%) patients belonged to very low, low, intermediate, high and very high-risk subgroup, respectively. Median follow-up of all patients was 16 months (range, 0.7–142.6 months). Patients proceeding to allogeneic stem cell transplantation (allo-SCT) were censored at transplantation. Excluding those patients, median follow-up was 23 months (range, 0.7–142.6 months). Data lock for this analysis was 15 July 2019. Detailed demographic and clinical characteristics are given in Table 1. All patients gave written informed consent according to Duesseldorf MDS registry and/or MDS biobank which were both approved by the local ethics committee (MDS-registry 3 973 and MDS-biobank 3768). In order to confirm the previously established assay specificity^17^ and furthermore to discriminate between healthy and malignant hematopoiesis we also analyzed samples of 12 healthy controls (HC) and of 17 non-MDS cytopenias diagnosed at our center with idiopathic cytopenia(s) of undetermined significance (ICUS) (*n* = 7), idiopathic thrombocytopenia (*n* = 2), toxic bone marrow failure (*n* = 2), renal anemia (*n* = 2), aplastic anemia (*n* = 2), anemia of chronic disease (*n* = 1) and cyclic neutropenia (*n* = 1) (supplementary Table 1).

**Table 1:** Patient and clinical characteristics

	No.	%
No.	94	
Median age, years (range)	61.5 (22–84)	
Gender		
Female	33	35
Male	61	65
Bone marrow blasts, median/range		4 (0–18)
Peripheral blasts, median/range		0 (0–11)
Leukocytes, median/range (/µl)	3,3 × 10^3^ (0,7–26 × 10^3^)	
Hemoglobin, median range (g/dl)	9,5 (4,8–14,8 10^3^)	
Platelets, median/range (/µl)	80 × 10^3^ (6–670 × 10^3^)	
WHO 2016 classification		
MDS del5q	7	7
MDS-U	3	3
MDS SLD	3	3
MDS RS SLD	1	1
MDS MLD	39	41
MDS EB1	16	17
MDS EB2	25	27
Primary MDS	86	91
Therapy related MDS	8	9
IPSS-R		
Very low	3	3
Low	28	31
Intermediate	28	30
High	14	14
Very high	21	22
Karyotype		
Normal	41	44
Abnormal	52	55
Complex	21	22
Missing	1	1
Cytogenetic risk group^a^		
Very good	4	4
Good	52	55
Intermediate	6	6
Poor	22	23
Very poor	9	10
Missing	1	1
Presence of certain molecular mutations^b^ (ASXL1, EZH2, TET2, TP53, DNMT3A, RUNX1)	24	26
Treatment		
Transfusion only	6	7
Growth factors	22	23
Lenalidomide	7	7
HMA	10	11
Intensive chemotherapy	5	5
Allogeneic stem-cell transplantation	44	47

*No*. number, *WHO* World Health Organization, *MDS SLD* MDS with single lineage dysplasia, *MDS RS* *SLD* MDS with ring sideroblasts and single lineage dysplasia, *MDS MLD* MDS with multilineage dysplasia, *MDS EB1* MDS with excess blasts 1, *MDS EB2* MDS with excess of blasts 2, *MDS del5q* myelodysplastic syndrome with isolated del(5q), *MDS-U* myelodysplastic syndrome unclassifiable, *HMA* hypomethylating agents

^a^According to IPSS-R

^b^Information based on results from clinical routine, but not on a comprehensive molecular analysis of all patients

### Quantitative assessment of PB cell WT1 expression

Quantitative assessment of *WT1*-mRNA expression in PB mononuclear cells was performed at diagnosis using the Ipsogen® WT1 ProfilQuant® Kit in accordance to manufacturers’ instructions as previously described^18^. Briefly, after RNA extraction using the RNeasy Mini Kit (Qiagen, Hilden, Deutschland) 1 μg RNA was reversely transcribed. cDNA was then subjected to RT-qPCR reaction using primers and probes in accordance with the manufacturers’ instructions. All experiments were carried out in duplicate on a Rotor-Gene Q 5plex HRM instrument ABL served as control gene and results are expressed as ratio of *WT1* copies per 10^4^ *ABL* copies. In line with the quality control requirements of the manufacturer results from samples containing <4 246 *ABL* copies were discarded. Based on results from a large cohort of diagnostic AML and healthy samples this ELN certified, standardized, plasmid-based assay uses a validated PB cut-off level of 50 *WT1* copies/10^4^ *ABL* copies to distinguish between normal and overexpression of *WT1-*mRNA^17^. Therefore, in our analysis values above this cutoff were judged as overexpression. First, peripheral blood *WT1*-mRNA expression level was correlated with clinical parameters such as MDS WHO subtype 2016 and IPSS-R risk categories. Next, for the purpose of this analysis patients were dichotomized based on the validated cut-off level into those with normal expression (defined as <50 *WT1* copies/10^4^ ABL copies) and those with overexpression of *WT1-*mRNA (defined as >50 *WT1* copies/10^4^ ABL copies) and subsequently compared regarding their outcome in terms of progression-free survival (PFS), leukemia-free survival (LFS) and overall survival (OS).

### Statistical analyses

OS was calculated as time from diagnosis to death from any cause or last follow-up in survivors. PFS was defined as time from diagnosis until (1) progression to a higher IPSS-R risk category, or (2) a higher subgroup according to WHO 2016, e.g., from non-blastic to blastic subgroup, (3) AML transformation or (4) death with those censored at last contact who were alive and had not progressed so far. All patients who underwent allo-SCT were censored at the time of allo-SCT. OS, PFS and LFS were estimated using Kaplan-Meier method using the log-rank test for univariate comparisons. For categorical variables frequencies were displayed and differences were estimated using cross tabulation and Fisher’s exact t-test as well as one-way ANOVA test, while for continuous variables medians (ranges) are given with the Mann-Whitney test employed to detect differences. Multivariate analysis was performed using the proportional hazard regression analysis (multiple Cox regression model). In all analyses, a *p*-value < 0.05 was considered to be statistically significant. Statistical analyses were performed using GraphPad Prism® 5.01 (GraphPad Software Inc., La Jolla, USA) and SPSS Statistic for Windows (SPSS Inc. Chicago, IL).

## Results

### Peripheral blood *WT1*-mRNA expression in patients with MDS according to WHO 2016 and IPSS-R

**Fig. 1:**
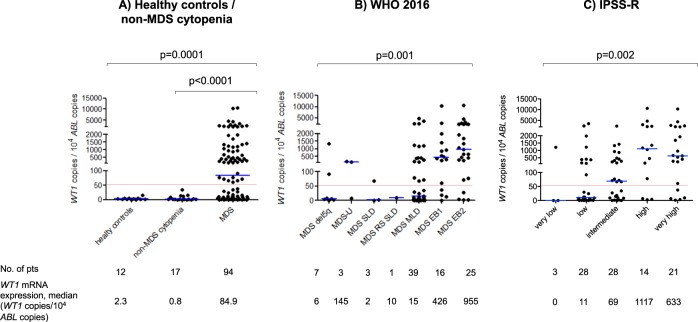
Peripheral blood *WT1*-mRNA expression level. **a** In patients with non-MDS cytopenia (*n* = 17) compared to patients with MDS and in 94 patients with MDS according to **b** WHO 2016 and **c** IPSS-R

Median PB *WT1*-mRNA expression level of all patients was 84.9 *WT1* copies/10^4^ ABL copies (range, 0 to 10 589 *WT1* copies/10^4^ ABL copies) (supplementary Fig. 1). Overall, 40 patients (43%) had normal PB *WT1*-mRNA expression at diagnosis (median 4.5 *WT1* copies/10^4^ ABL copies, range 0–37.5 *WT1* copies/10^4^ ABL copies), whereas 54 patients (57%) showed overexpression of *WT1*-mRNA (median 759 *WT1* copies/10^4^ ABL copies, range 61-10589 *WT1* copies/10^4^ ABL copies) (supplementary Fig. 2). Thereby, PB *WT1-*mRNA overexpression enabled significant discrimination between MDS and HC (0/12, 0%, *p* < 0.0001) as well as between MDS and non-MDS cytopenias (0/17, 0%, *p* < 0.0001, see also Fig. 1a). In MDS patients, PB *WT1-*mRNA expression level strongly correlated with disease category according to WHO 2016 classification as indicated both by the frequency of patients with *WT1*-mRNA overexpression in the respective entities (MDS SLD: 1/3 patients, 33%; MDS MLD: 16/39 patients, 41%; MDS EB1 12/16 patients, 75%; MDS EB2: 21/25 patients, 84%; *p* = 0.003) and by the median PB *WT1*-mRNA expression level within each subcategory (MDS SLD: 1.8 *WT1* copies/10^4^ ABL copies; MDS MLD: 15 *WT1* copies/10^4^ ABL copies; MDS EB1 1: 426.4 *WT1* copies/10^4^ ABL copies; MDS EB2: 954.5 *WT1* copies/10^4^ ABL copies; *p* = 0.001, Fig. 1b; supplementary Fig. 3). In addition, PB *WT1*-mRNA expression significantly correlated with IPSS-R risk categories, again indicated by the frequency of patients with *WT1-*mRNA overexpression (IPSS-R very low: 1/3 patients, 33%; IPSS-R low: 10/28 patients, 36%; IPSS-R intermediate: 16/28, 57%; IPSS-R high: 11/14 patients, 79%; IPSS-R very high: 16/21, 76%; *p* = 0.02) as well as by the median *WT1-*mRNA expression level within the respective subcategories (IPSS-R very low: 0.0 *WT1* copies/10^4^ ABL copies; IPSS-R low: 10.8 *WT1* copies/10^4^ ABL copies; IPSS-R intermediate: 69.1 copies *WT1* copies/10^4^ ABL copies; IPSS-R high: 1117 *WT1* copies/10^4^ ABL copies; IPSS-R very high: 632.7 *WT1* copies/10^4^ ABL copies; *p* = 0.002, Fig. 1c; supplementary Fig. 4).

### Correlation of PB *WT1*-mRNA expression level with hematologic parameters

Next, we correlated PB *WT1* expression with several hematologic parameters (Table 2). *WT1-*mRNA overexpression was significantly associated with decreased platelet and WBC count as well as with an increase of BM blast count (*p* = 0.04, 0.03 and 0.001, respectively), while no correlation with hemoglobin level was found (*p* = 0.46). Additionally, the presence of blasts in PB (*p* = 0.002) as well as an aberrant or complex karyotype (*p* = 0.02 and *p* = 0.03) were significantly linked to PB *WT1-*mRNA overexpression.

**Table 2:** Correlation of *WT1*-mRNA expression and hematologic parameters

	WBC (x10^9^/L)		Hb (g/dl)		PLT (×10^9^/L)		PB blast count (%)		BM blast count (%)		Age (years)	
*WT1* < 50*	3.85 (0.7–17)	**0.0345**	9.8 (4.8–14.8)	0.4616	103 (9–540)	**0.0425**	0 (0–5)	**0.0025**	3 (0–17)	**0.0013**	63.5 (45–84)	**0.0089**
*WT1* > 50*	2.7 (0.7–26)		9.3 (6–13)		67 (6–670)		0 (0–11)		6 (1–18)		59 (22–77)	

	Gender (female/male)			Karyotype (normal/abnormal)			Karyotype (normal/komplex)			PB blasts (presence/absence)		
*WT1* < 50^a^	12	28	0.3923	23	16	**0.0198**	23	5	**0.0296**	1	37	**0.0023**
*WT1* > 50^a^	21	33		18	36		18	15		13	34	

^a^Copies/10^4^ *ABL* copies; values are presented as medians with ranges

*p*-value < 0.05 was considered to be statistically significant

*No*. number, *WBC* white blood cell count, *Hb* hemoglobin, *PLT* platelet count, *PB* peripheral blood, *BM* bone marrow

### Prognostic impact of PB *WT1*-mRNA expression level in patients with MDS

**Fig. 2:**
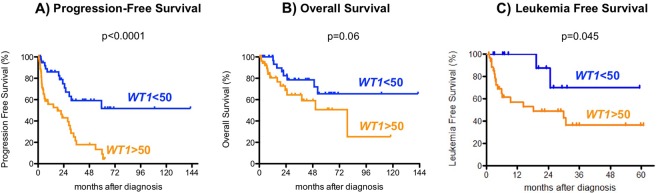
Outcome of patients with MDS based on *WT1*-mRNA expression. **a** Progression free survival (PFS), **b** overall survival (OS), and **c** leukemia free survival (LFS)

**Fig. 3:**
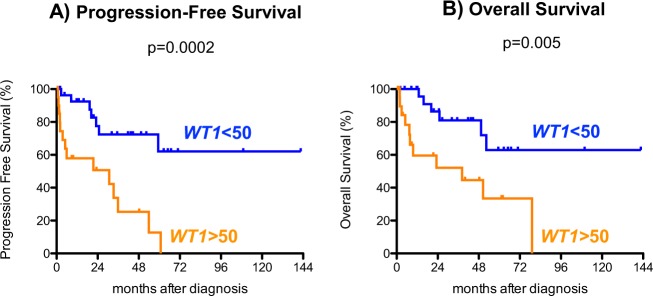
Outcome of patients with MDS based on *WT1*-mRNA expression excluding those patients proceeded to transplant. **a** Progression free survival (PFS) and **b** overall survival (OS)

Median PFS, LFS and OS for all patients were 28.9 months (range, 0.8–142.6 months; estimated 5-year PFS 27%, 95% CI: 14–39%), 30.8 months (range, 0.8–60.8 months; estimated 5-year LFS 44%, 95% CI: 27–64%) and 79.1 months (range, 0.8–142.6 months; estimated 5-year OS 58%, 95% CI: 44–74%) (supplementary Fig. 5). By comparing patients based on the *WT1-*mRNA expression status (overexpression, >50 *WT1* copies/10^4^ ABL copies vs. normal expression, <50 *WT1* copies/10^4^ ABL copies), we found that patients with *WT1-*mRNA overexpression had significantly higher frequency of disease progression compared to those with normal *WT1-*mRNA expression (*WT1-*mRNA overexpressing patients: 37 of 54 patients = 68.5% vs. patients with normal *WT1-*mRNA expression: 13 of 40 patients = 32.5%; *p* = 0.001). Accordingly, PFS was significantly lower in patients with *WT1-*mRNA overexpression in comparison to those with normal *WT1-*mRNA expression (median PFS: 18.2 months vs. not reached, *p* < 0.0001). Also the rate of patients who transformed into AML was higher in those exhibiting a *WT1-*mRNA overexpression compared to patients with normal pB *WT1-*mRNA expression (patients with normal *WT1-*mRNA expression: 2 of 13 patients, 15% vs. *WT1-*mRNA overexpressing patients: 18 of 37 patients, 48.6%; *p* = 0.0498). Consequently, LFS was significantly lower in patients with pB *WT1-*mRNA overexpressing compared to those with normal *WT1-*mRNA expression (median LFS: 18.2 months vs. not reached, *p* = 0.045). Regarding OS, we saw a similar trend in favor for patients with normal *WT1-*mRNA expression compared to those exhibiting pB *WT1-*mRNA overexpression, but without reaching statistical significance (median OS not reached vs. 79.10, *p* = 0.06) (Fig. 2). The differences regarding PFS and OS were statistically significant after excluding patients who underwent allo-SCT during the course of disease (Fig. 3). PB *WT1-mRNA* expression level on PFS retained its independent prognostic value also in multivariate analysis (*p* = 0.0001, Table 3).

**Table 3:** Prognostic impact of *WT1*-mRNA expression on outcome of patients with MDS in multivariate analysis

	Progression-free survival HR (95% CI)	*p*-value	Overall survival HR (95% CI)	*p*-value
*WT1-*mRNA expression status (<50 vs. >50^a^)	0.239 (0.152–0.566)	0.0001	0.486 (0.208–1.136)	0.096
IPSS-R (very low/low vs. int/high/very high)	0.640 (0.325–1.258)	0.196	1.222 (0.875–1.705)	0.240

^a^Copies/10^4^ *ABL* copies

### PB *WT1*-mRNA expression status can predict PFS in low and intermediate risk MDS

**Fig. 4:**
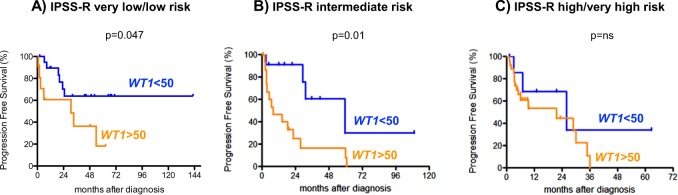
Progression free survival (PFS) of IPSS-R subgroups based on *WT1*-mRNA expression. **a** IPSS-R very low (*n* = 3) and low (*n* = 28) risk MDS, **b** intermediate risk MDS (*n* = 28), and **c** high (*n* = 14) and very high (*n* = 21) risk MDS

Having demonstrated that *WT1-*mRNA expression status correlated with IPSS-R category as well as survival and progression in the entire cohort, we then examined whether *WT1-*mRNA expression status may also refine the prediction of prognosis (OS and PFS) within each IPSS-R risk categories separately. Due to limitations regarding patient numbers in the very low and high-risk subgroups patients within these categories were summarized with patients of low and very high-risk group, respectively. Hereby, it became apparent that in the very low/low and intermediate risk subcategories PFS significantly differed between patients showing normal PB *WT1-*mRNA expression (IPSS-R very low/low, median PFS: not reached; IPSS-R intermediate, median PFS: 59.4 months) compared to patients exhibiting PB *WT1-*mRNA overexpression (IPSS-R very low/low, median PFS: 30.8 months; IPSS-R intermediate, median PFS: 7.8 months) (*p* = 0.047 for IPSS-R very low/low and *p* = 0.01 for IPSS-R intermediate, respectively; Fig. 4a, b). In contrast, no impact of *WT1-*mRNA expression status on PFS was found in patients within the IPSS-R high/very high-risk categories (Fig. 4c). Furthermore, no impact on OS was observed within any IPSS-R defined risk group. Overall, these data implied that in addition to a general prognostic impact in patients with MDS, *WT1-*mRNA expression seemed to refine the prediction of disease progression particularly in patients with IPSS-R very low/low and intermediate risk.

## Discussion

Using a standardized ELN-certified assay we here showed that the transcription factor *WT1* was overexpressed on mRNA level in 57% of patients with MDS and that PB *WT1-*mRNA overexpression strongly correlated with disease categories and risk stages according to WHO 2016 classification and IPSS-R respectively. Furthermore, our data indicated that PB *WT1-*mRNA expression status significantly correlated with prognosis of MDS patients with those patients showing *WT1-*mRNA overexpression having a higher risk for disease progression and AML transformation and accordingly shorter progression-free, leukemia-free and overall survival. This prognostic impact of PB *WT1-*mRNA expression was independent of the IPSS-R as confirmed by multivariate analysis. In further support of this, *WT1-*mRNA expression status enabled a more precise prediction of prognosis in terms of PFS in patients within the IPSS-R very low/low and intermediate risk groups.

Persisting cytopenias and signs of dysplasia in the BM are prerequisites to establish the diagnosis of MDS. Still, in particular if dysplastic features are subtle it is sometimes difficult even for trained hematologists to distinguish between MDS and reactive cytopenias or cytopenias related other bone marrow syndromes such as aplastic anemia^19^. Furthermore, even the detection of gene mutations such as DNMT3A, ASXL1, and TET2 may not be sufficient enough to accurately diagnose MDS, since these mutation can be found in approximately 10% of healthy individuals older than 65 years without evidence for a hematological malignancy summarized as “clonal hematopoiesis of indeterminate potential” (CHIP) as well as in patients with aplastic anemia^20,21^. In our analysis PB *WT1-*mRNA overexpression was found in 57% of patients with MDS, while the remaining 43% had normal *WT1*-mRNA expression. Still, *WT1*-mRNA overexpression nicely enabled discrimination between MDS and and non-MDS cytopenias, and this effect applied when looking at all MDS, but also when focusing on those with a BM blast count <5% (*WT1-*mRNA overexpression in non-MDS cytopenia 0/17 and *WT1-*mRNA overexpression in MDS < 5% BM blast count 26/53, *p* = 0.0001, see also supplementary Fig. 6). These data are in line with two previous reports^22,23^ and indicate that measurement of PB *WT1-*mRNA expression may serve as an easy accessible marker and helpful tool to differentiate between MDS and non-MDS cytopenias. In those patients with a definitive diagnosis of MDS it has been previously shown using an in-house assay that *WT1-*mRNA expression level, which was determined in BM in the majority of patients, correlated with disease category according French American Britain (FAB) classification and IPSS risk categories^11^. In our analysis, we confirm and expand this strong correlation of *WT1-*mRNA expression level measured in the PB to the WHO 2016 classification and to IPSS-R. For instance, in our cohort PB *WT1-*mRNA overexpression was detected at diagnosis in 68% of the patients with intermediate-, high- or very high risk according to IPSS-R, who generally represent potential candidates for allo-SCT. In contrast to this, individual mutations of the most frequently affected genes such as TET2 or ASXL1, which may be accessible for tracking by mutation-specific techniques, are present in only about 20 to 35% of patients. These findings suggested a greater informativeness and applicability of *WT1-*mRNA expression for MRD monitoring after allo-SCT. Indeed, supporting its clinical usefulness we and others have recently demonstrated that monitoring of *WT1-*mRNA expression levels in PB allows sensitive and specific detection of MRD after allo-SCT in a large proportion of MDS patients^17,18,24^. Furthermore, monitoring of disease and response kinetics by PB *WT1-*mRNA expression to guide therapeutic decisions may also be used in MDS patients treated with other therapies such as hypomethylating agents^25^ or proteasome inhibitors^11,26^.

Besides its value as diagnostic tool and MRD marker a limited number of reports suggested a prognostic impact of *WT1-*mRNA expression level on outcome of patients with MDS. However, interpretation and translation of these results into clinical practice has been hampered by a limited number of patient samples, varying sample sources (PB vs. BM) and the use of different assays without comparable cut-off levels^13–16^. Still, it would be of interest to correlate PB and BM *WT1*-mRNA expression levels in paired samples, a point we were not able to perform in our analysis due to insufficient number of paired BM samples. As a specific strength we here used a commercially available, ELN-certified assay, which offered a validated cut-off level to discriminate between normal and *WT1-*mRNA overexpression and thereby enabled reproducible and comparable analysis of *WT1-*mRNA expression in a standardized manner across different laboratories. Another advantage of this assay is that it facilitates measurement of *WT1-*mRNA in with a greater sensitivity and specificity than in BM thereby offering patient comfort.

In addition, our results showed that PB *WT1-*mRNA expression at diagnosis represented a new prognostic factor in MDS patients which was independent from the IPSS-R and exhibited additional prognostic information. This was true when analysing the entire MDS cohort (PFS, LFS), but also enabled us to more precisely predict PFS in patients with IPSS-R very low/low and intermediate risk. Even though patients may fall in one of the same of these categories their course of disease may vary individually ranging from indolent conditions with near normal life expectancy to those with rapid progression to AML. Thus, refinement of the prognosis by the addition of *WT1-*mRNA expression status may help to better stratify patients and tailor surveillance strategies or even support individual treatment decisions, for example to consider allo-SCT at an earlier time point.

A similar approach like ours to improve IPSS-R-based risk stratification by integrating molecular markers is the subject of an on-going initiative of the IWG-PM which tries to incorporate the prognostic information of somatic mutations into the IPSS-R. Results reported so far showed that inclusion of gene mutations improved assessment of prognosis in MDS^7–10^. However, they also indicated that screening procedures for somatic mutations is a complex issue, integrating numerous mutations, e.g., up to 13 in the analysis of the IWG-PM^27^, and is thereby time- and resource-intensive. In addition, the limited access to molecular diagnostics at least in some regions, lack of standardized analyses and reporting, the need of BM as optimal sample source and the limited frequency of individual mutations also hampers the broad application of gene mutation testing in the entire MDS population. Given the intrinsic limitation of a retrospective analysis information on molecular aberrations at primary diagnosis was only available for a limited proportion of patients impeding inclusion of molecular data into uni- and/or multivariate outcome analyses.

In summary, measurement of PB *WT1-*mRNA expression as single molecular marker using a standardized, ELN-certified assay with reproducible cut-offs represented a clinically valuable, patient friendly and resource-effective supplementation in diagnostic work up and refinement of prognostic information provided by the IPSS-R.

## Supplementary information

Supplementary Figures

Supplementary Figure Legend

Supplementary Table 1

Supplementary Table Legend
