# Supplementary material for: Prognostic impact of peripheral blood WT1-mRNA expression in patients with MDS
Source: Blood Cancer J. 2019 Nov 12;9(11):86. doi: 10.1038/s41408-019-0248-y (PMC6851368; doi:10.1038/s41408-019-0248-y)
Supplement: Supplementary file 1 — Supplementary Figures [file 41408_2019_248_MOESM1_ESM.pptx]

## Slide 1
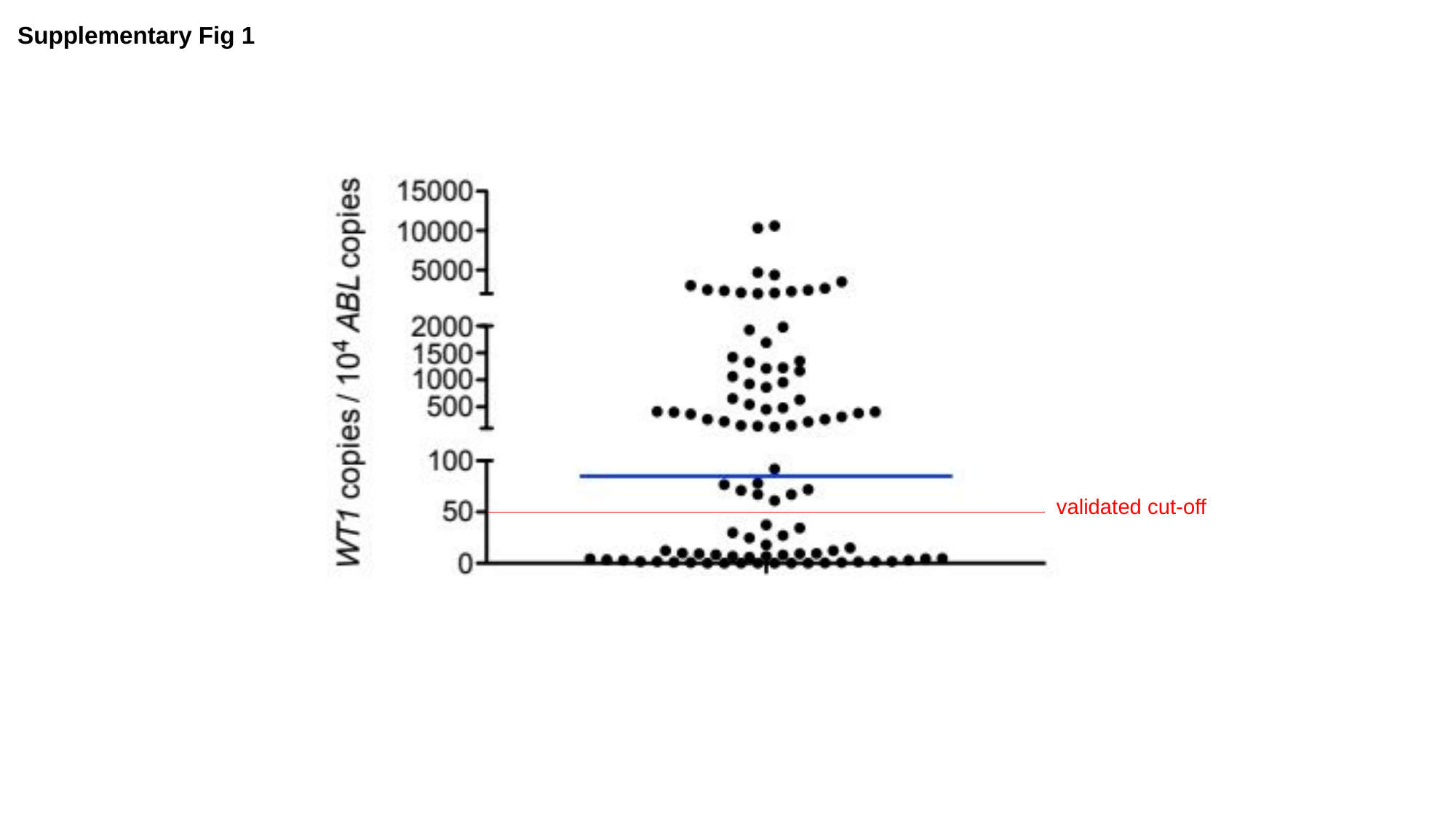

Supplementary Fig 1
validated cut-off

## Slide 2
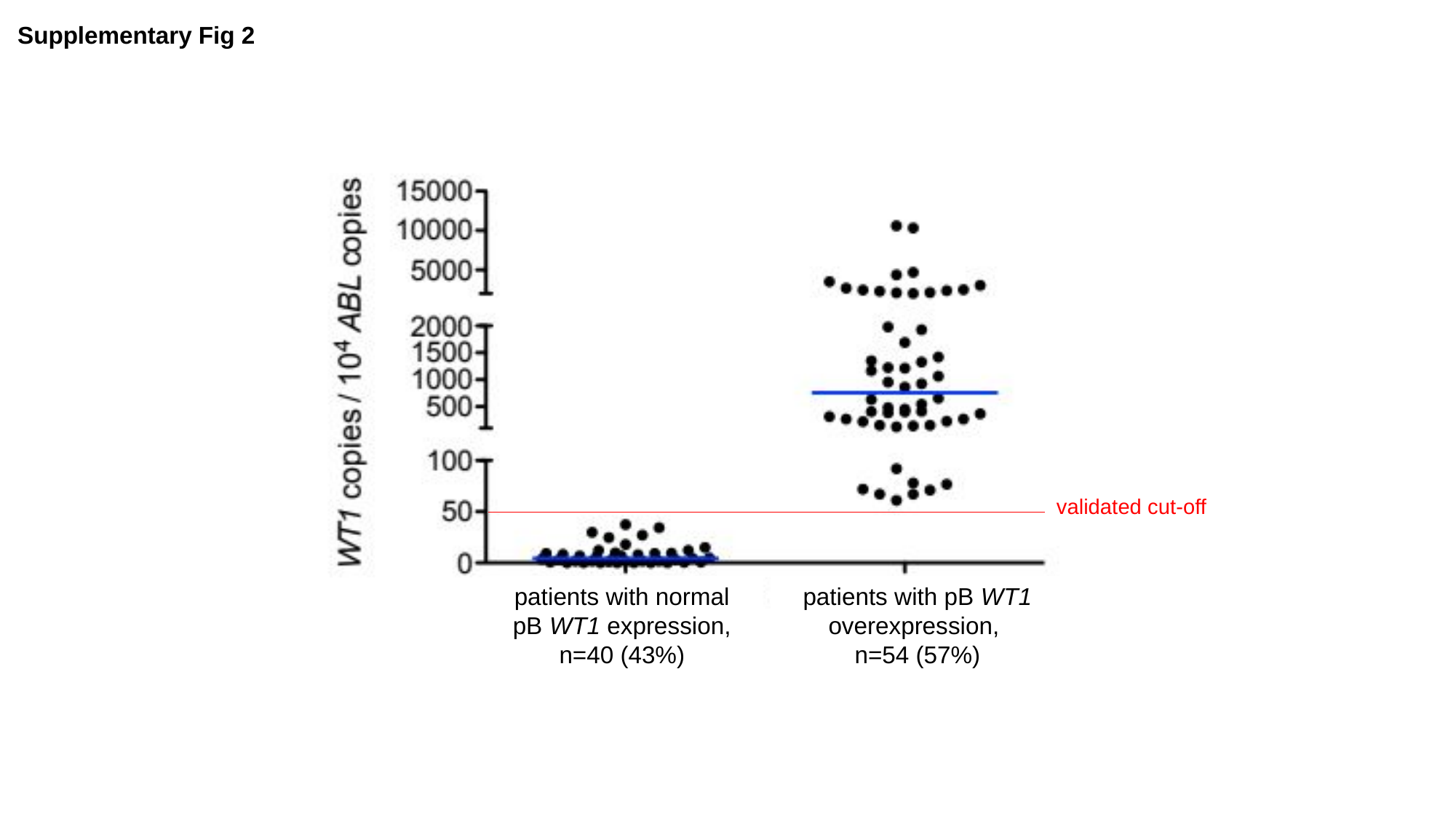

Supplementary Fig 2
validated cut-off
patients with normal pB WT1 expression, n=40 (43%)
patients with pB WT1 overexpression,
n=54 (57%)

## Slide 3
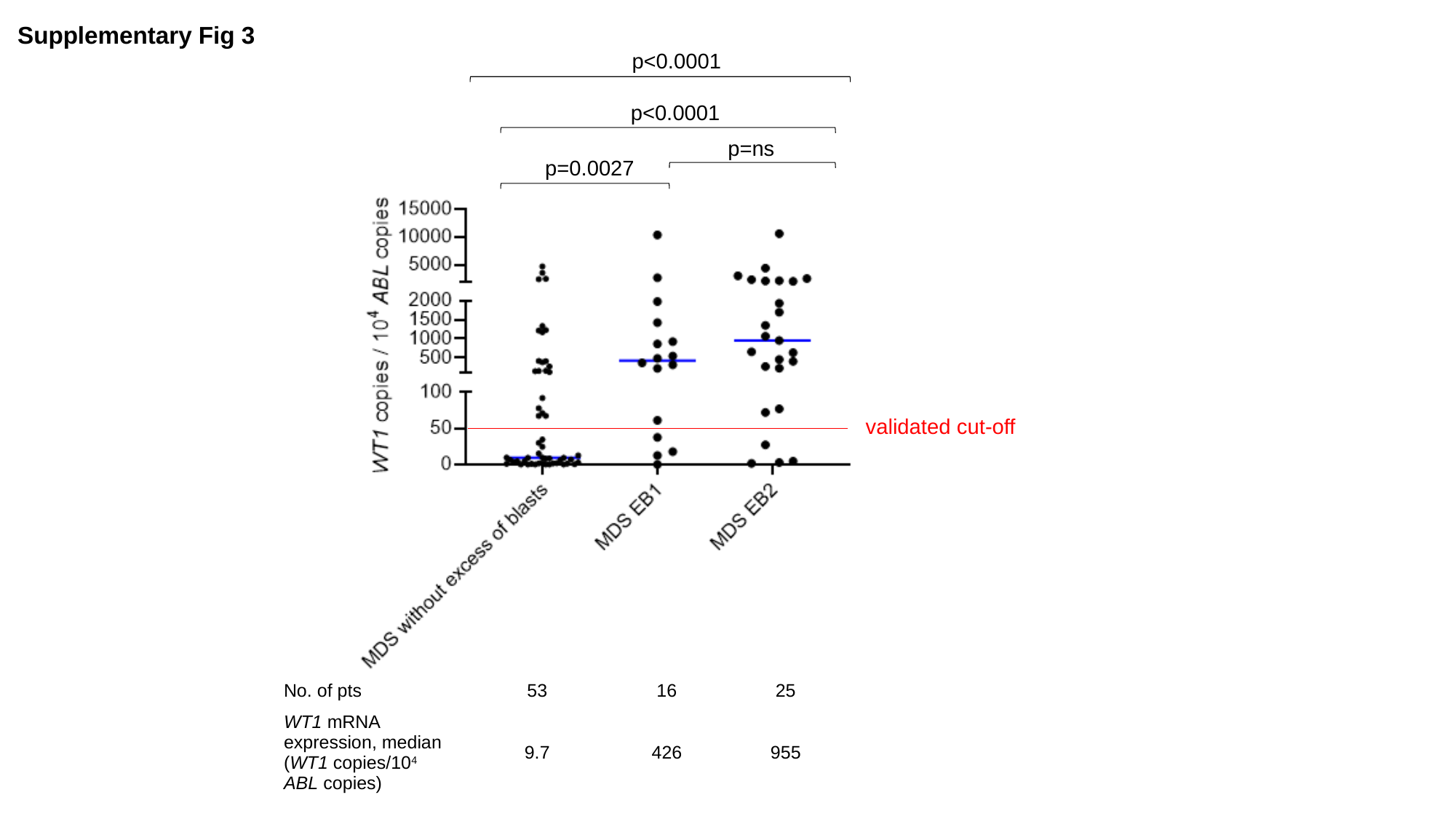

Supplementary Fig 3
p<0.0001
p<0.0001
p=ns
p=0.0027
validated cut-off
| No. of pts | 53 | 16 | 25 |
| --- | --- | --- | --- |
| WT1 mRNA expression, median (WT1 copies/104 ABL copies) | 9.7 | 426 | 955 |

## Slide 4
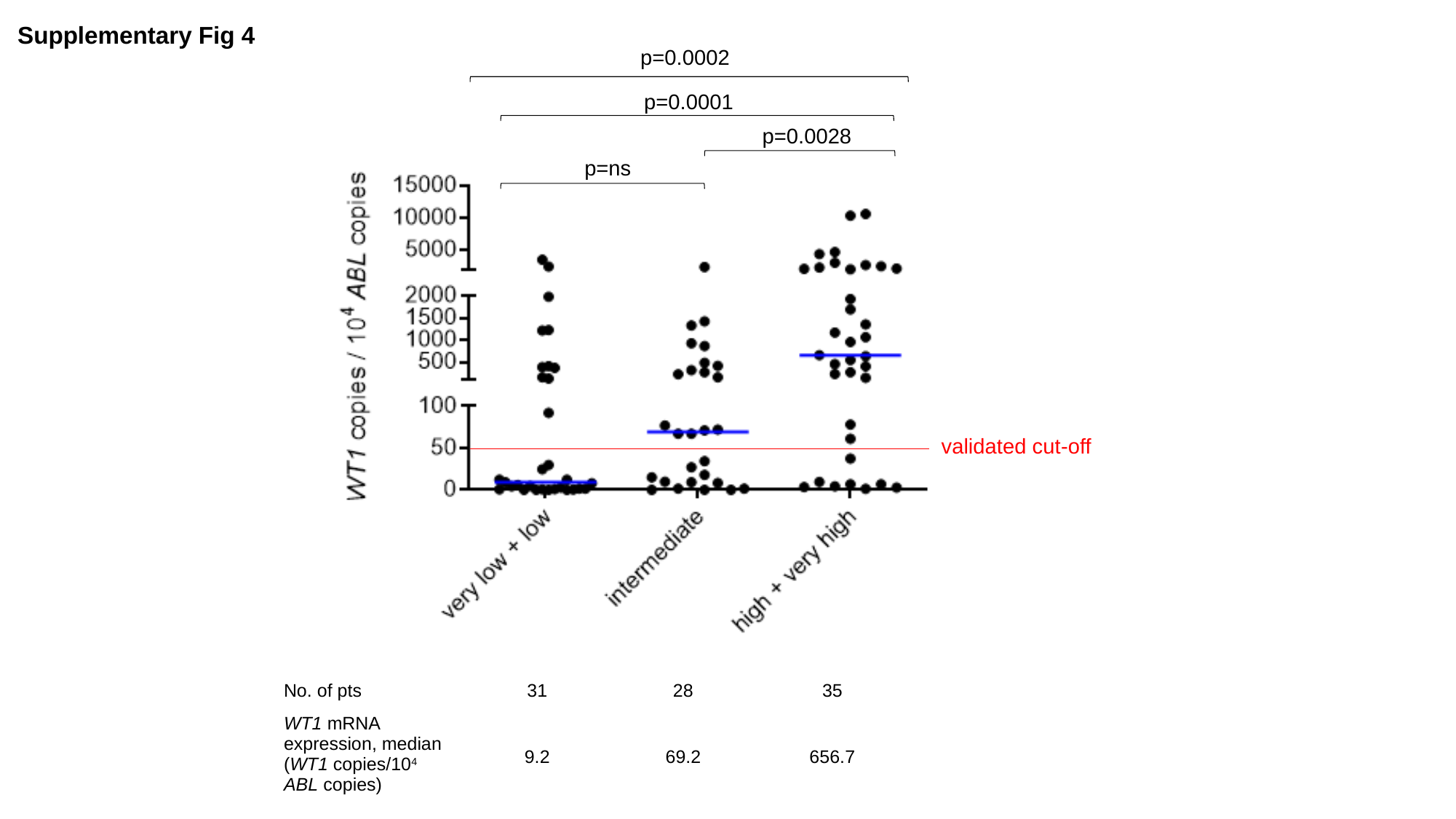

Supplementary Fig 4
p=0.0002
p=0.0001
p=0.0028
p=ns
validated cut-off
| No. of pts | 31 | 28 | 35 |
| --- | --- | --- | --- |
| WT1 mRNA expression, median (WT1 copies/104 ABL copies) | 9.2 | 69.2 | 656.7 |

## Slide 5
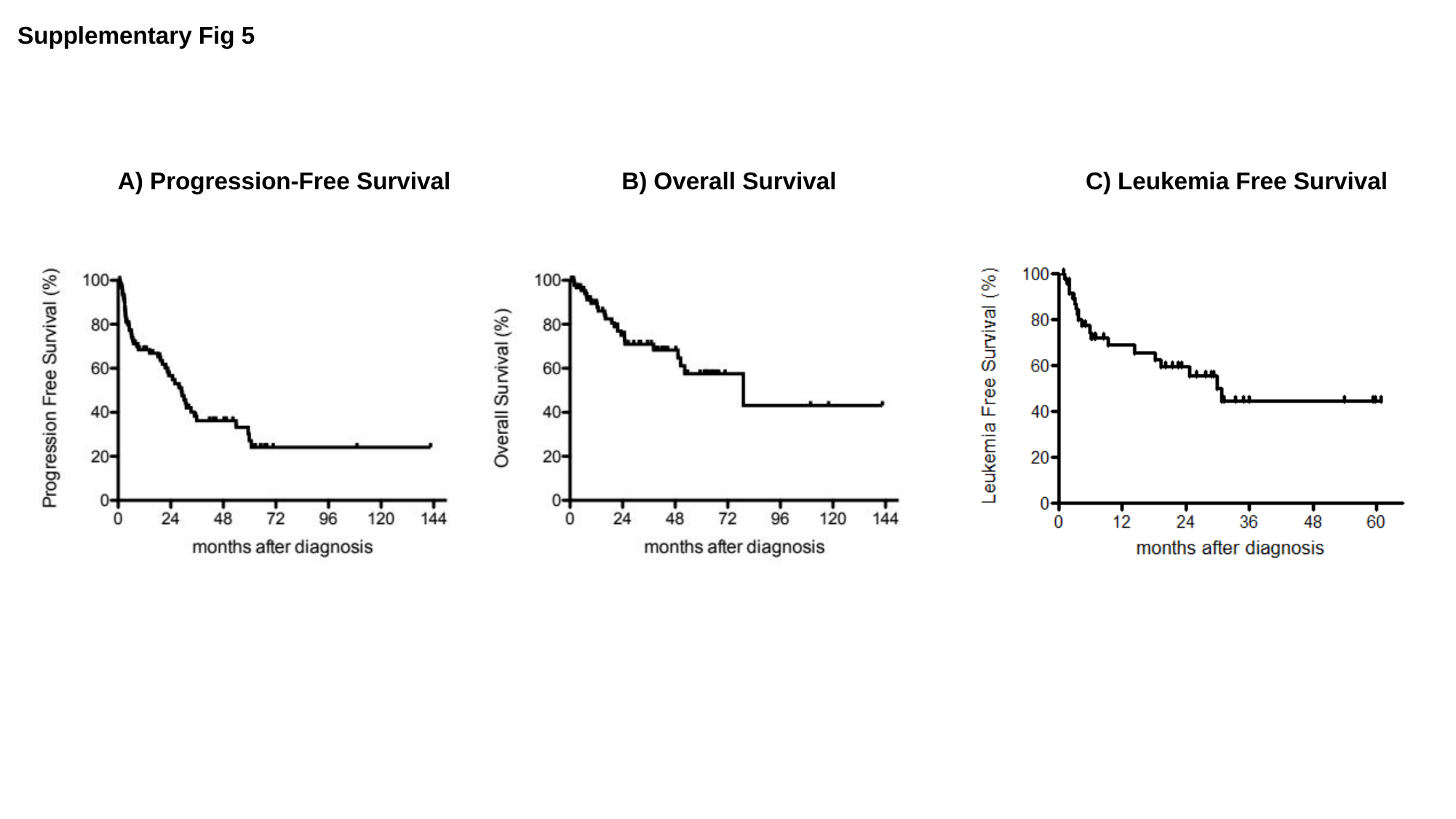

Supplementary Fig 5
A) Progression-Free Survival
B) Overall Survival
C) Leukemia Free Survival

## Slide 6
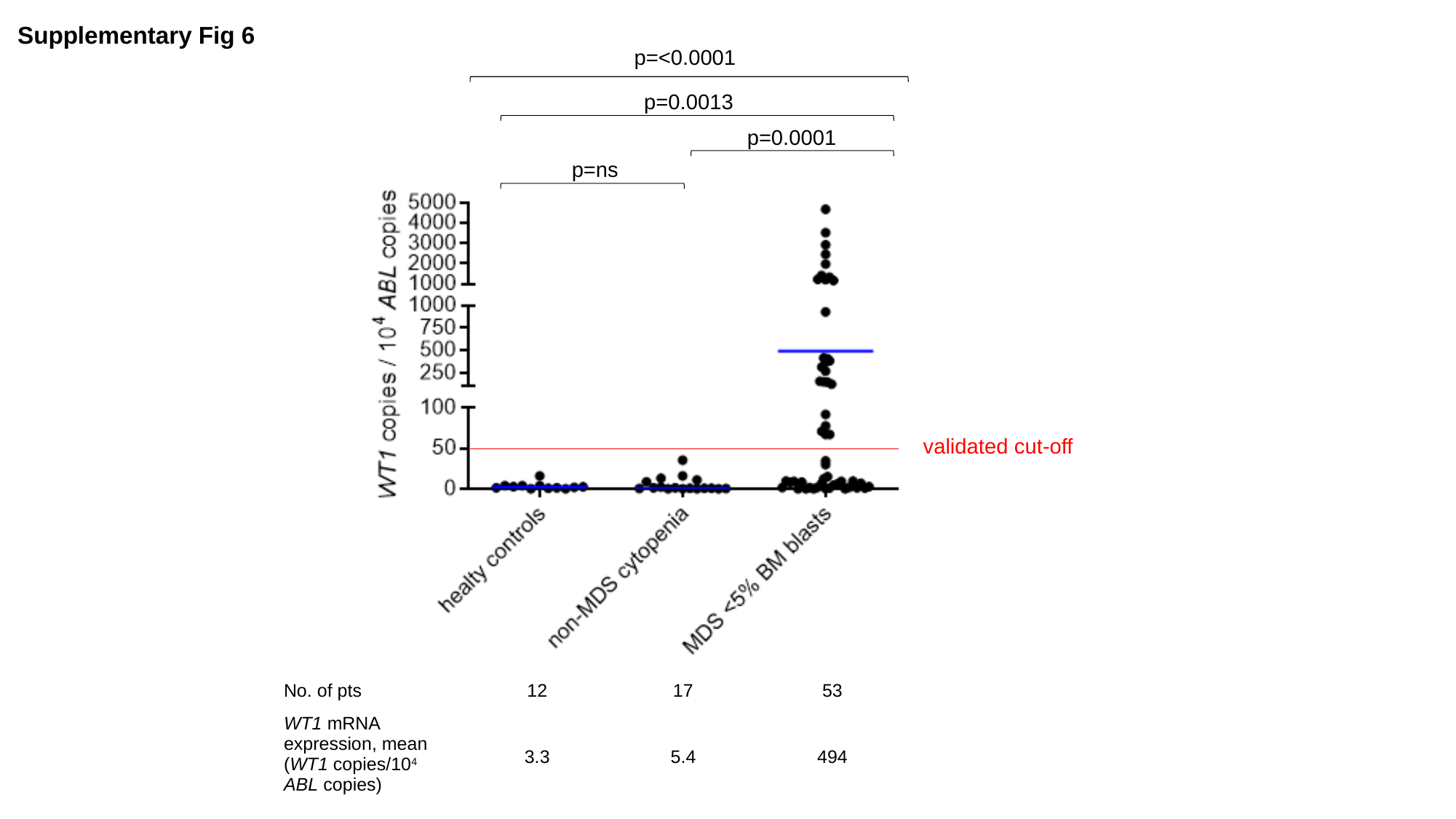

Supplementary Fig 6
p=<0.0001
p=0.0013
p=0.0001
p=ns
validated cut-off
| No. of pts | 12 | 17 | 53 |
| --- | --- | --- | --- |
| WT1 mRNA expression, mean (WT1 copies/104 ABL copies) | 3.3 | 5.4 | 494 |
