# Supplementary material for: Prognostic impact of peripheral blood WT1-mRNA expression in patients with MDS
Source: Blood Cancer J. 2019 Nov 12;9(11):86. doi: 10.1038/s41408-019-0248-y (PMC6851368; doi:10.1038/s41408-019-0248-y)
Supplement: Supplementary file 2 — Supplementary Figure Legend [file 41408_2019_248_MOESM2_ESM.docx]

Supplemental Figure 1: *WT1* mRNA expression level detected per quantitative real-time polymerase chain reaction in peripheral blood of 94 patients with MDS. Median pB *WT1* mRNA expression level was 84.9 *WT1* copies/10^4^ *ABL* copies (range, 0 – 10589 *WT1* copies/10^4^ *ABL* copies) indicated by the blue line. The red line indicates a validated cut-off of level 50 *WT1* copies/10^4^ *ABL* copies to distinguish between normal expression (<50 *WT1* copies/10^4^ *ABL* copies) and overexpression (>50 *WT1* copies/10^4^ *ABL* copies) of *WT1* mRNA in peripheral blood.

Supplemental Figure 2: *WT1* mRNA expression level detected per quantitative real-time polymerase chain reaction in peripheral blood of 94 patients with MDS. Forty patients (43%) showed a normal pB *WT1* mRNA expression level (left scatter dot plot; median 4.5 *WT1* copies/10^4^ ABL copies, range 0-37.5 *WT1* copies/10^4^ ABL copies), whereas 54 patients (57%) exhibited a pB *WT1* overexpression (right scatter dot plot; median 759 *WT1* copies/10^4^ ABL copies, range 61-10589 *WT1* copies/10^4^ ABL copies). Median pB *WT1* expression level in each group is indicated by the blue line. The red line indicates a validated cut-off of level 50 *WT1* copies/10^4^ *ABL* copies to distinguish between normal expression (<50 *WT1* copies/10^4^ *ABL* copies) and overexpression (>50 *WT1* copies/10^4^ *ABL* copies) of *WT1* mRNA in peripheral blood.

Supplemental Figure 3: Peripheral blood *WT1* mRNA expression level in 94 patients with MDS according to WHO 2016 disease categories. Patients without excess of blasts (MDS del5q, MDS-U, MDS SLD, MDS RS SLD, MDS MLD) were summarized and compared to patients with MDS EB1 and patients with MDS EB2, respectively. Median pB *WT1* mRNA expression level is indicated by the blue line. The red line indicates a validated cut-off of level 50 *WT1* copies/10^4^ *ABL* copies to distinguish between normal expression (<50 *WT1* copies/10^4^ *ABL* copies) and overexpression (>50 *WT1* copies/10^4^ *ABL* copies) of *WT1* mRNA in peripheral blood.

Supplemental Figure 4: Peripheral blood *WT1* mRNA expression level in 94 patients with MDS according to IPSS-R. Due to limitations regarding patient numbers in the very low and high risk subgroups patients within these categories were summarized with patients of low and very high risk group, respectively. Median pB *WT1* mRNA expression level is indicated by the blue line. The red line indicates a validated cut-off of level 50 *WT1* copies/10^4^ *ABL* copies to distinguish between normal expression (<50 *WT1* copies/10^4^ *ABL* copies) and overexpression (>50 *WT1* copies/10^4^ *ABL* copies) of *WT1* mRNA in peripheral blood.

Supplemental Figure 5: (a) Progression Free (PFS), (b) Overall Survival (OS) and (c) Leukemia Free Survival (LFS) in 94 patients with MDS. Median PFS, LFS and OS were 28.9, 30.8 and 79.1 months after diagnosis.

Supplemental Figure 6: Peripheral blood *WT1* mRNA expression level in healthy controls (n=12) and patients with non-MDS cytopenia (n=17) compared to patients with MDS and <5% bone marrow blasts (n=53). Mean pB *WT1* mRNA expression level is indicated by the blue line. The red line indicates a validated cut-off of level 50 *WT1* copies/10^4^ *ABL* copies to distinguish between normal expression (<50 *WT1* copies/10^4^ *ABL* copies) and overexpression (>50 *WT1* copies/10^4^ *ABL* copies) of *WT1* mRNA in peripheral blood.
