# Supplementary material for: Prognostic impact of peripheral blood WT1-mRNA expression in patients with MDS
Source: Blood Cancer J. 2019 Nov 12;9(11):86. doi: 10.1038/s41408-019-0248-y (PMC6851368; doi:10.1038/s41408-019-0248-y)
Supplement: Supplementary file 3 — Supplementary Table 1 [file 41408_2019_248_MOESM3_ESM.docx]

*Supplementary Table 1: Peripheral Blood WT1-mRNA expression level in healthy controls and non-MDS cytopenia*

| **diagnosis** | **peripheral blood *WT1*-mRNA expression level** |
| --- | --- |
| aplastic anemia | 0.6 |
| aplastic anemia | 16 |
| anemia of chronic disease | 0 |
| cyclic neutropenia | 35.3 |
| idiopathic cytopenia of undetermined significance* | 1.4 |
| idiopathic cytopenia of undetermined significance* | 0.8 |
| idiopathic cytopenia of undetermined significance* | 0.5 |
| idiopathic cytopenia of undetermined significance* | 11 |
| idiopathic cytopenia of undetermined significance* | 8.8 |
| idiopathic cytopenia of undetermined significance* | 0.5 |
| idiopathic cytopenia of undetermined significance* | 0.6 |
| idiopathic thrombocytopenia | 0 |
| idiopathic thrombocytopenia | 0 |
| toxic bone marrow failure | 0 |
| toxic bone marrow failure | 1.3 |
| renal anemia | 13.1 |
| renal anemia | 2.1 |
| healthy control | 2.7 |
| healthy control | 0.2 |
| healthy control | 1.4 |
| healthy control | 0.8 |
| healthy control | 4.0 |
| healthy control | 2.0 |
| healthy control | 1.4 |
| healthy control | 4.0 |
| healthy control | 3.9 |
| healthy control | 0.0 |
| healthy control | 2.5 |
| healthy control | 16.1 |

* regarding those patients who were diagnosed with idiopathic cytopenia(s) of undetermined significance (ICUS) no mutations potentially classifying them as clonal cytopenia of undetermined significance (CCUS) were detected
