# Supplementary material for: Prognostic impact of peripheral blood WT1-mRNA expression in patients with MDS
Source: Blood Cancer J. 2019 Nov 12;9(11):86. doi: 10.1038/s41408-019-0248-y (PMC6851368; doi:10.1038/s41408-019-0248-y)
Supplement: Supplementary file 4 — Supplementary Table Legend [file 41408_2019_248_MOESM4_ESM.docx]

Supplementary Table Legend

Supplemental Table 1: Peripheral Blood *WT1*-mRNA expression level in healthy controls and non-MDS cytopenia

* regarding those patients who were diagnosed with idiopathic cytopenia(s) of undetermined significance (ICUS) no mutations potentially classifying them as clonal cytopenia of undetermined significance (CCUS) were detected
